# Diet and physical activity changes among low-income families: perspectives of mothers and their children

**DOI:** 10.1080/17482631.2019.1658700

**Published:** 2019-08-27

**Authors:** Jenny Zhen-Duan, Bery Engebretsen, Helena H. Laroche

**Affiliations:** a Department of Psychology, University of Cincinnati, Cincinnati, OH, USA; b Primary Healthcare Inc, Urbandale, IA, USA; c Department of Internal Medicine, University of Iowa, Iowa City, IA, USA

**Keywords:** Diet, family, obesity, parent-child relations, physical activity

## Abstract

**Purpose**: The current study explored how mothers and their children influence each other’s diet and physical activity.

**Methods**: We conducted semi-structured interviews with women with diabetes and their children (*N* = 18) from eight low-income families.

**Results**: Two approaches to changes emerged: collaborative and non-collaborative. Families using collaborative approaches believed they could sustain positive changes through accepting family changes, encouragement, abstaining from buying certain foods, modelling and compromise. Within families using non-collaborative approaches, some challenges included using more individualistic approaches and poor communication. Lack of information and resource constraints challenged all families.

**Conclusion**: Interventions should reinforce family collaborative approaches and teach skills for families to work together towards a healthier lifestyle.

## Introduction

The prevention of childhood obesity is a national public health priority in the USA, as approximately 37% of adults and 17% of children are obese (Flegal, Kruszon-Moran, Carroll, Fryar, & Ogden, 2016; Ogden et al., 2016). Obese adults are more likely to have children at risk for obesity (Reilly et al., 2005; Sonneville et al., 2012; Whitaker, Jarvis, Beeken, Boniface, & Wardle, 2010). Furthermore, children living below the poverty line are disproportionately at risk to become obese when compared to children in higher income homes (Singh, Siahpush, & Kogan, 2010). Many dietary and physical activity behaviours are learned and reinforced within the context of the family and could be altered to improve health outcomes (Holm, Wyatt, Murphy, Hill, & Odgen, 2012; Reicks et al., 2015; Scaglioni, Arrizza, Vecchi, & Tedeschi, 2011; Sonneville et al., 2012). In fact, family-focused programmes have been shown to be efficacious and cost-effective in treating obesity among both adults and children (Epstein et al., 2014). However, childhood obesity prevention has often focused on schools, with little attention on family-based obesity prevention (Wang et al., 2013). To this end, the design of effective family-based obesity prevention interventions will require more understanding of the complex interactions between parents and children around food and physical activity.

## Social Cognitive Theory

The Social Cognitive Theory (SCT) posits that certain principles guide behaviour for health promotion, including individual characteristics such as knowledge about health, perceived self-efficacy, and outcomes expectations (Bandura, 2004). Knowledge about health might include information about adequate diet and exercise and has been linked to greater childhood obesity prevention (Davison, Jurkowski, Li, Kranz, & Lawson, 2013). Self-efficacy is defined as the individual’s confidence in their ability to achieve a targeted behaviour (Baranowski & Hearn, 1997). For example, higher self-efficacy has been linked to greater weight loss, and healthier eating among adults and youth (Anderson-Bill, Winett, & Wojcik, 2011; Anderson, Winett, & Wojcik, 2007; Annesi, Johnson, & McEwen, 2015; Fitzgerald, Heary, Kelly, Nixon, & Shevlin, 2013; Larsen, McArdle, Robertson, & Dunton, 2015). Outcome expectation is defined as what the individual expects to happen if they modify their behaviour (Baranowski & Hearn, 1997). Thus, individuals are more likely to adopt behaviours that they believe will produce positive outcomes and disregard ones that they believe will lead to undesirable outcomes (Anderson et al., 2007; Bandura, 2001; Larsen et al., 2015).

Aside from individual factors, another key aspect of the SCT model is the inclusion of the collective agency (Taylor, Baranowski, & Sallis, 1994). As Albert Bandura described, “people do not operate as isolates” (Bandura, 2004). In a more general sense, communities are interconnected, have shared beliefs in their collective efficacy, and work together to improve lives of all members (Bandura, 2004). Family members also represent a collective agency that share a set of knowledge, motivation, and skillsets that could work together towards a common goal of improving their lives (Bandura, 2001). The idea of reciprocal determinism highlights that factors such as individual characteristics, the environment, and the actual behaviour will constantly interact and influence each other to predict future behaviours. For example, a person’s committed action towards losing weight might be influenced by the interaction of individual characteristics (e.g., self-efficacy), the environment (e.g., family support), and the behaviour itself (e.g., restricted caloric intake motivates future restrictions). The family model of reciprocal determinism (Baranowski & Hearn, 1997) emphasizes the collective agency of the family unit and how family members interact to influence each other’s environment, individual characteristics and behaviour. In this way, a family member’s health behaviour both influences and is influenced by the behaviour of other family members (Baranowski & Hearn, 1997; Taylor et al., 1994).

## Parents’ influence on children

Consistent with the SCT model, the literature shows parents’ behaviours can directly affect their children’s knowledge about health, self-efficacy, and outcome expectations. Parents who share their knowledge about diet and activity could influence their children’s knowledge and outcome expectations (Kakinami, Houle-Johnson, & McGrath, 2016). Similarly, parental modelling of behaviour for their children results in children learning new skills and having increased motivations (e.g., outcome expectations) around health behaviours (Dwyer et al., 2017; Hughes, O’Connor, & Power, 2008; Joyal-Desmarais et al., 2019). In line with the concept of reciprocal determinism, parents may also influence child behaviours through changing the physical or social environment. For example, parents affect children’s diet by controlling availability of foods, making healthy foods easily accessible, and creating structure around mealtimes (Fulkerson, Story, Neumark-Sztainer, & Rydell, 2008; Han et al., 2015; Ong, Ullah, Magarey, Miller, & Leslie, 2017; Reicks et al., 2015). Aside from diet, parents can promote physical activity in their child by taking them to the park or driving them to sports practice (Carver, Timperio, Hesketh, & Crawford, 2010; Ghekiere et al., 2016; Sukys, Majauskienė, Cesnaitiene, & Karanauskiene, 2014). Alternatively, parents could limit children’s active play outside because of their concerns about neighbourhood safety (Carver, Timperio, & Crawford, 2008; Carver et al., 2010; Datar, Nicosia, & Shier, 2013; Veitch et al., 2017). Parents also influence family normative expectations about what family members should eat (Reicks et al., 2015) and the degree to which children should be physically active (Joyal-Desmarais et al., 2019).

## Children’s influence on parents

Although it is known that children also influence the shared environment and parent’s health behaviours, this topic has received less research attention. Studies show parents’ diets are higher in saturated fat (Laroche, Wallace, Snetselaar, Hillis, & Steffen, 2012), parents are more likely to gain weight over time (Laroche et al., 2013; Umberson, Liu, Mirowsky, & Reczek, 2011), and are less active (Berge, Larson, Bauer, & Neumark-Sztainer, 2011), compared to non-parents, possibly through the direct or indirect influence of having a child in the home. Children’s food preferences often weigh heavily on the selection of family food purchasing and consumption, (Wingert, Zachary, Fox, Gittelsohn, & Surkan, 2014) highlighting the strong influence children have on the family’s diet. Aside from food choices, children may also indirectly influence the environment by shaping finances and time availability. Time spent caring for children could also limit time allotted for cooking and shopping (Beshara, Hutchinson, & Wilson, 2010) and being physically active (Gaston, Edwards, Doelman, & Tober, 2014; Hull et al., 2010; Rhodes et al., 2015). Particularly among low-income families, the cost of raising children could strain family finances leaving less resources to buy certain foods and pay for opportunities to be physically active (Burke et al., 2017).

Children also have more direct influences on parents’ diet and physical activity. Children can play active roles in being a part of and advocating for, change in diet and physical activity for parents with diabetes (Gadhoke, Christiansen, Swartz, & Gittelsohn, 2015; Laroche et al., 2009). Some parents may be motivated to be more physically active to be good role models for their children, to provide opportunities for their children to be physically active, or to stay healthy and be able to care for their children for the long term (Mailey, Huberty, Dinkel, & McAuley, 2014), possibly impacting their outcome expectations for being more active. Therefore, interventions that do not consider, or take advantage of, the child’s influence may be missing other effective tools for promoting change.

## The current study

The current study uses a family SCT framework, with an emphasis on the family model of reciprocal determinism, to explore how adults and their children influence each other’s diet and physical activity. Only a handful of studies have simultaneously examined adult and children’s perspectives, but these have mainly focused on diet, one included physical activity and one focused on sedentary time. For example, these studies have emphasized parents’ roles in promoting healthy diet (Williams, Veitch, & Ball, 2011), different dietary practices among low-income families with an adult with diabetes (Laroche, Davis, Forman, Palmisano, & Heisler, 2008), lack of healthy diet information among Latino parents and children (Lilo, Munoz, & Cruz, 2018), why children engage in sedentary behaviours from a parent and child perspective (Hidding, Altenburg, van Ekris, & Chinapaw, 2017), and ways children can act as change agents among six Native American families (Gadhoke et al., 2015). Though these are important additions to the obesity prevention literature, none specifically addressed children’s interest in making changes for themselves or other family members and how their desires influenced family behaviour. The current paper addresses the dearth of literature by including both adults’ and their children’s perspectives on strategies used to manage their own diet and physical activity, and how these strategies affect other family members. By including perspectives of low-income adults and their children, we are better able to build models of behavioural change for family-based, obesity prevention and intervention programmes among high-risk groups.

## Method

### Study design and sample

We followed the principles of community-based participatory research, which emphasize community involvement throughout the research process (Israel, Eng, Schulz, & Parker, 2005). We partnered with health workers employed in a community health centre that served patients, where 91% of adults had incomes below 200% of the federal poverty line and approximately 35% were obese. Our partners in the health centre were a part of the study design, interview content, recruitment, and final data interpretation. In addition, another partner organization that provided food to local food pantries helped the research team with recruitment. To gather information-rich cases, we sampled participants based on the adult members’ age (21–30, 31–40, 41 and up), gender, and race/ethnicity (Latino, African American, Caucasian) (Patton, 2002). We purposefully sampled adults in waves from the community health centre and the local food pantries to fulfil our sampling framework. The research team reviewed transcripts, codes and family summaries for new themes or repetitions of prior themes. When 13 families were sampled, the research team concluded that they were still gaining new perspectives and continued recruitment. After 21 families were sampled, the research team concluded that later interviews echoed issues from earlier interviews (thematic saturation) and recruitment ended.

Adults were eligible to participate in the study if they lived with a minor and if they had glucose intolerance, diabetes, or were obese (Body Mass Index of 30 and above). If the participating adult was not the primary food preparer in the home, we interviewed the primary food preparer separately. The children of the participating adults were also recruited if they were between 10 and 17 years old. One child was randomly chosen among those available to be interviewed (except in one case where a second child requested to also join the interview). All adults and children were required to speak either English or Spanish. Although the main project sample included 21 families, the current study only included the eight families whose children were also eligible to be interviewed. The remaining 13 adults were parents whose children were not within the recruitment age or not available for interview. Table I illustrates the breakdown of the eight families included in this study and demographic descriptors of all participants (*N* = 18), including eight mothers, one grandmother, and nine children. Seventy five percent of these families answered at least 1 question positively on the food insecurity scale. Coincidentally, all the participating adults in this subset of families had diabetes. Pseudonyms were used for all families and features that were felt to be identifiable were left out or altered to create a more generalized description.

**Table I.** Demographic characteristics of participating families and individuals.

Units	Family (*N* = 18)	Participant	Sampling description	Sex	Age	Education level
1	Lopez (n = 2)	Mother Lopez	Target adult and is the primary food preparer	Female	54	8 years or less
		Child Lopez		Female	17	
2	Jones (n = 2)	Mother Jones	Target adult and is the primary food preparer	Female	39	Some college
		Child Jones		Male	17	
3	Rivera (n = 2)	Mother Rivera	Target adult and is the primary food preparer	Female	38	8 years or less
		Child Rivera		Female	11	
4	Torres (n = 3)	Mother Torres	Target adult and is the primary food preparer	Female	37	Some college
		Child #1		Female	16	
		Child #2		Male	10	
5	Rodriguez (n = 2)	Mother Rodriguez	Target adult and is the primary food preparer	Female	52	8 years or less
		Child Rodriguez		Female	15	
6	Family Smith (n = 3)	Mother Smith	Target adult	Female	34	NA
		Grandmother Smith	Primary food preparer	Female	65	
		Child Smith		Male	16	
7	Family Park (n = 2)	Mother Park	Target adult and is the primary food preparer	Female	40	Some high school
		Child Park		Male	17	
8	Family Brooks (n = 2)	Mother Brooks	Target adult and is the primary food preparer	Female	42	Completed college
		Child Brooks		Female	13	

### Procedure

Qualitative, semi-structured interviews were conducted because this methodology allows researchers to further explore participants’ lived experiences through follow-up questions (Whiting, 2008). All semi-structured interviews were done face-to-face by a trained, Masters-level, native Spanish-speaking bilingual interviewer. An interview guide was used that included open-ended questions to engage participants in a rich discussion. In general, adults were asked about (1) changes they would like to or have made to their diet and physical activity; (2) facilitators and barriers to those changes; (3) resources they need to help them make changes; (4) desire to make changes to their children’s diet; and (5) facilitators and barriers to improving their children’s diet and activity. Adult interviews lasted from 45 to 120 minutes (most were around 70 minutes). Children were interviewed without the adult present to gather the most honest opinions. Child interviews included questions about (1) activities they participated in with their family; (2) where they ate with and without their family; (3) current physical activities; (4) changes their parent needs to make to be healthy, (5) diet or activity changes they would like to make; (6) barriers and facilitators to change. The study was approved by the Institutional Review Board of the University of Iowa.

### Data analysis

Interviews were audio-taped, transcribed, and verified. Data were analysed and collected concurrently so insights from completed interviews could inform ongoing data collection and analysis. We analysed the interviews thematically, assisted by Max QDA, a qualitative data analysis software. Our overall approach followed the “Editing Analysis Style,” which contained both deductive and inductive elements (Crabtree & Miller, 1999). Deductive codes were derived from analysis frameworks based on the SCT and literature review. Inductive codes were derived from the interviews to create new categories and refine framework categories.

Two doctoral students transcribed all interviews, verbatim, while retaining all verbal/audio information from the recordings. The same doctoral students independently coded selected portions of the transcripts and used an iterative process to compare results until coding agreements were reached. The principal investigator read selected transcripts to compare her code interpretations with the student coders and served as a third decision maker for any disagreements. After the coding scheme was developed, the remaining interviews were divided between the two doctoral students who coded them independently. Additionally, the content of each interview was summarized by the two students to highlight context and issues for each family and dynamics between family members. The combination of coded interviews and family summaries allowed us to look across and within families. We increased the credibility of our conclusions through rigorous and systematic data collection and analysis, the use of multiple readers and coders, and by documenting the reasons for analytic decisions (Mason, 2002).

## Results

Participating families highlighted changes they made to improve their health that were consistent with previous studies, such as serving smaller food portions, eliminating junk food, and going on walks in the park. Aside from general strategies, adults and children described how they interacted with each other to influence health behaviours (i.e., reciprocal determinism). The following sections will highlight the novel contribution from our paper by describing the two general approaches to changes (i.e., collaborative and non-collaborative) the families in our study engaged in when approaching diet and physical activity changes. Of note, some families took a collaborative approach to some changes and not others. We will also highlight examples of parent's and children's perspectives on how they influence change in one another.

### Collaborative

When families used a more collaborative approach to behaviour changes, they engaged in more open communication and mutual support between family members to create a positive health change. Families working collaboratively reported a stronger belief in their abilities to create and sustain the health change, related to self-efficacy, a cornerstone of the of the SCT framework (Bandura, 2004). Some factors influencing collaborative approaches included (a) accepting changes (especially children), (b) encouraging each other, (c) abstaining from buying or eating certain foods, (d) leading by example, and (e) mutual expectations for changes and compromise.

#### Accepting changes

In these cases, the mother felt she helped the family by making healthy changes and the process was facilitated by the children and other family members’ openness to changes rather than resisting. Family Lopez is a Latino family, which included Mother Lopez, her husband, their two children and one grandchild. Mother Lopez explained that she had been serving smaller food portions and cooking healthier meals. She explained that diet changes had been accepted by family members as they understood that improving her diet would improve her diabetes control:

Mother Lopez: I started changing everything. I believe that [these changes help my children a lot] because my children like what I do a lot … For example, if I fry a chicken, before I would fry it with skin and all, right, all of it—like this with grease. Not anymore. Now I remove [the skin] … they like it a lot. (translated from Spanish)

An important strategy Mother Lopez described using was the ability to culturally-adapt traditional Latino foods but still ensure that they were healthy, such as making Mexican vegetable and beef stew. In her interview, Child Lopez agreed that she and her siblings had responded well to the dietary changes initiated by their mother and believed that the family was healthier.

#### Encouraging each other

In these examples, mothers encouraged children to be healthier but additionally children believed they had a role in encouraging their mother to be healthier. The Lopez family supported and encouraged each other around diet and physical activity. Mother Lopez described that her children helped sustain her motivation and enthusiasm to continue eating healthier and exercising more often. Both her children encouraged her to work out and would often join her in these workouts:

Mother Lopez: The girl takes me to Zumba. Sometimes she stays with me

Interviewer: This helps you to, to stay active?

Mother Lopez: Yes

Interviewer: And when you don’t want to go? Does she encourage you or not?

Mother Lopez: Yes (chuckles). [My son told me] “Let’s go [for a walk]” … yesterday—he told me “Mommy, if you can’t go for a walk, don’t go.” I told him, “But I need to go for a walk. I don’t want to stay here …” If I stop going, I’m regretting not going—

Interviewer: … and when you do go—

Mother Lopez: I feel good (translated from Spanish)

Family Park included a Caucasian woman, African American husband, and their three teenage children. Even though they struggled to work collaboratively on consuming a healthier diet, they worked together to improve physical activity behaviours. In his interview, Child Park believed that he played a role in improving his mother’s health because he accompanied her on walks and motivated her to engage in more physical activity.

#### Abstaining from buying and eating certain foods

Participants described children and spouses avoiding purchasing or consuming certain foods to help other family members eat healthier (especially the mother with diabetes). For example, Child Lopez said that he avoided eating before bed so that his mother is not tempted:

Child Lopez: [I think I influence her eating behaviours] Maybe she saw the food and she’d want some. Like, she’ll crave it.

Another example is Family Jones, which included an African American woman who had recently lost over 100 pounds, and her son. Child Jones described facilitating healthy dietary changes by not buying unhealthy foods that would tempt Mother Jones:

Child Jones: Oh yeah, I do [believe I have a part in her eating because] I see something at the store that I probably want and I just be like “mom can I get this?” She’ll be like “you know it’s not good for you …” Like chips, like—pretty much chips and pop. [But I don’t buy it because] then she’ll join in and probably drink some pop or whatever.

Family Rivera is another Latino family comprised of the Rivera parents and their four children. Mother Rivera described that Father Rivera helps by supporting the new dietary changes in many ways, including encouraging their children to eat out less as a family:

Mother Rivera: My husband says that … it’s a way to look out for me … so I’m not tempted. I see them eating hamburgers and pizzas—so he tells the children ‘so that your mom doesn’t have eat alone, [let’s not eat] them. (translated from Spanish)

#### Leading by example

Family members described a reciprocal process of positive behavioural changes through modelling. Especially mothers were motivated to make changes in order to be an example for their children and influence their behaviour. Mother Jones has led by example and has in turn affected her son’s behaviour by engaging him in the positive changes:

Mother Jones: You know, he actually follows me and now I actually got him hooked on water … He actually likes water now … because like you know, all I did was, every time he opened the fridge, all he saw was pop. Pop. Kool aid, … juice … and now that I drink more water, he drinks more water.

Mother Rivera explained that changing her attitudes towards physical activities also benefited her children because she used to become upset if she had to walk long distances. However, she described that she pushed herself to be more active and now took the children to the park, walked them to the bus stop, and parked far away when shopping so they all have to walk.

#### Mutual expectations and compromise

Another key element was that when mothers set diet or exercise expectations for their children, the children also expected the mothers to follow the same rules. In addition, compromise between parents and children regarding food choices and expectations made health change easier. For example, Mother Rivera stated that her children were eating healthier foods since she prohibited them from eating too much junk food. Child Rivera, in turn, described that she encouraged her mother to eat healthier by asking her to eat less candy:

Children Rivera: [I think Mom] can start eating like healthy, like healthy fruits, to make her more active and make her have more strength … [I try to help her with that]—like to tell her to stop eating chocolate.

Another family described their work towards becoming collaborative and setting mutual expectations. Family Brooks is comprised of an African American woman, her mother (i.e., Grandmother), and a child. Mother Brooks described a lot of problems reconciling the family diet because of different food preferences regarding vegetable intake:

Mother Brooks: … [It would be easier] if everybody would eat it with me and not complain about it and if I could take the time to cook a nice meal … [I have tried] to add more vegetables in our meals and I try to do that

Interviewer: How well does, does it go over?

Mother Brooks: (both chuckle) Not very well … My mom likes canned vegetables. I prefer the frozen. And my daughter tends to be picky when it comes to eating vegetables … Every once in a while [a strategy works]. We had some low-fat fajitas the other night and that worked really well!

Mother Brooks explained that she had often tested recipes and compromised with the other two family members to find recipes everyone liked. Mother Brooks mentioned many examples where she found it difficult to be fair to both her mother and daughter regarding meal choices. However, they worked together to find common ground through trial and error in the pursuit of improving dietary practices in the household. Several other families in our study were successful in improving diet by communicating, planning, and compromising.

### Non-collaborative

When families took a non-collaborative approach to a behaviour change, one or all family members expressed their desire to improve the health behaviour but each approached it individually. Two main themes come up within a non-collaborative approach, including (a) individualistic approaches and (b) poor communication.

#### Individualistic approaches

In these families, all or some family members viewed changed as focused on the individual, often the adult with diabetes, rather than endorsing change as something to be done as a family. Family Torres is an example in which all family members expressed wanting to make changes in diet and exercise in themselves and other family members. However, the process was viewed as an individual-effort rather than a collective-effort. Family Torres is Latino family comprised of Mother Torres, her husband and their three children, two of whom participated in the interview (ages 16 and 10). Mother Torres described encountering challenges when she tried to implement healthier eating around the household. She perceived that her children resisted by expressing that they were not “sick” with diabetes and thus did not need to make changes:

Mother Torres: We have tried putting a piece of salmon … on their plate, rice, and vegetables … well the only thing that disappears is the rice and [the children don’t want to eat healthy] because it’s all about mom, “cause mom’s the one that’s sick,” so the changes that are good for me might not be the good changes that they want for themselves. Even though we’re trying, they don’t like it.

Similarly, Mother Park explained that her children avoided eating healthy foods when Father Park did not eat them: (mimicking voice) ‘“dad doesn’t eat green beans, that’s why I don’t’, or ‘dad, don’t eat lettuce, that’s why I don’t eat it.”’ Family Park is another example of a family where the children believed that Mother was the only one that needed to make changes as she was the one with diabetes. Even though members of Family Park worked collaboratively towards physical activity goals, this family had more difficulty working collaboratively on an improved diet. Child Park recognized that his mother needed to eat healthier but did not advocate this for himself:

Child Park: We’ve been eating fast food [and she needs to change that] because … well, she already has diabetes so I don’t want it to get worse [because] she could die.

However, Mother Park stated that it is difficult to eat healthy at home because of individualistic approaches to change: “nobody else in the house likes to eat anything healthy.”

Another case following an individualistic approach to positive health changes was Family Rodriguez, a Latino family where both Mother and Father have diabetes. The Rodriguez household was comprised of the parents, their three children, and one grandchild. Even though the family as a whole wanted to improve dietary behaviours, the interviewed child was the only one engaging in more proactive health behaviours. Child Rodriguez stated that she wished her parents would increase their vegetable intake and their physical activity. However, she went to the gym several times a week without any family members. Child Rodriguez also described being involved in some of the positive changes in the house when her parents were not around, including preparing healthy foods for herself and for her younger siblings.

#### Poor communication

Some families that worked non-collaboratively did not clearly communicate their diet and exercise plans, even though their goals were aligned. For example, Child Torres 1 shared several of her mother’s concerns, such as being overweight, lacking self-confidence, and worrying about having diabetes in the future. Child Torres 1 had implemented some individual strategies in the past:

Child Torres 1: I would like to change myself—I’m overweight, I don’t like it, I would like to change so I can be … more, like self-confident in a way. [I need to start] eating less, stop eating rice, maybe more vegetables—stop eating sweets, maybe drink water more often—I tried it, it helped until like winter came in then everything completely went down. [Because] you’re stuck at home, you have nothing to do and all you see is something sweet and you’re like “oh, yay” … [I need to] involve myself in a sport. ‘Cause it would be better for my health so I won’t [have] diabetes. Sometimes I try to force myself to doing something … so—then I won’t have to put insulin or take medicine.

In contrast, Mother Torres perceived that Child Torres 1 did not care about her well-being and resisted eating healthier. Thus, Mother Torres prepared two different meals, one for adults and another for her children:

Mother Torres: The others [the children] don’t really care right now about—They don’t really worry about their physical being unless you remind them … I mean, she knows she’s putting on weight and she knows how heavy she is, she knows that she is not moving around, she knows what she’s suppose to be doing … Even though we’re trying [to eat healthier] they [the children] … they don’t like it. Most times [I have to prepare two different meals], it’s the same thing except that me and my husband have extras like vegetables, pickled cabbages … me and him are always eating vegetables and something else besides what we’re eating for dinner.

Child Torres 2 was younger and more limited in his responses. Nonetheless, both children expressed that they believed their mother needed to exercise more but perceived that their mother “doesn’t want to,” is busy, or does not feel well. Mother Torres, on the other hand, expressed that even though she wanted to exercise more, she is busy balancing caretaking responsibilities and college classes. Thus, even though there are places where their goals align, they are not communicating about how they might work together.

### Barriers for all families

Aside from the two general approaches described, most individuals in our study described barriers commonly experienced by low-resourced families, such as: (a) lack of information and (b) resource constraints.

#### Lack of information

Families lacked information on healthy diet and recipes, physical activity options and how to present new foods to children. One explanation for why Mother and Father Rodriguez were unable to engage in the same positive diet and physical activity changes as their daughter might be a lack of information. Mother Rodriguez said she did not know what she needed to do for herself or what to do to improve her family’s diet and requested more information on healthy diet and recipes. She also said she does not engage in dietary restrictions but has decreased the amount of lard used during meal preparation. In contrast, Child Rodriguez perceived herself as being knowledgeable about healthy diet and said that she received information from the school:

Child Rodriguez: [I have received information about diabetes from my school]—… every month they do a program where … instead of making hot [foods], they make us eat like vegetables and all that. And we do more exercise. We take like one class off to go out to PE and do exercise.

It is possible that Child Rodriguez has been more proactive about engaging in healthy behaviours because of the information provided by the school, whereas Mother Rodriguez has limited access to health information. Language barriers possibly contributed to the lack of information because Mother Rodriguez’s primary language is Spanish. Lack of information was a recurring theme in both Spanish and English-speaking households. For example, both Mother Torres and Mother Park expressed great desire to improve their diet but also expressed frustration around not knowing what “eating better” meant.

Both Mother Torres and Mother Park explicitly shared that they have had to apply poor strategies to encourage their children to eat healthier. For example, they used threats to try to get their children to eat better: “if you don’t eat right, you’re getting diabetes.” Mother Park described that one of her daughters is a “hypochondriac” and will not eat anything that can hurt her. Therefore, she told her daughter that soda has sugar and dyes that cause cancer so she stopped drinking it. Both Mother Torres and Mother Park shared that lack of information on how to encourage their children to eat healthy made them resort to threats.

Lack of information was a also prominent issue raised by families already implementing healthy dietary habits, such as Family Lopez described above. Mother Lopez explained that although she had made positive diet changes within the household, she was often confronted with questions about whether foods were beneficial or detrimental:

Mother Lopez: It’s difficult to follow a healthy diet because—sometimes I am very motivated to make a healthy meal for myself but sometimes I don’t know what I’m supposed to be eating and what I shouldn’t. I think to myself—if it’s too high in fat, I shouldn’t eat it—grains, that I can’t eat either.

Mother Lopez described an experience that was commonly observed in families engaging in collaborative and non-collaborative approaches. Specifically, some participants made changes with limited information and others found it overwhelming to make dietary changes due to lack of information.

#### Resource constraints

One challenge faced by most of these families was a lack of resources related to chronic poverty; this made it difficult to work towards a healthier lifestyle. Family Smith is an example of the many contextual challenges that low-income families face when trying to make healthy changes in their household. Family Smith is comprised of a Latina woman, her Caucasian mother, two children, and another child relative. Grandmother Smith is the primary food preparer and described that she could not change the food she prepared because choices in food are inflexible due to a very limited income:

Grandmother Smith: I guess I haven’t made a whole lot of changes because … personally I don’t see that there’s a lot of room for change cause … I try to do the best I can with what I got, but I seem to fail a lot.

Interviewer: And you say that, why?

Grandmother Smith: Because things don’t stretch, it doesn’t stretch.

Interviewer: And stretch meaning in the amount that you get?

Grandmother Smith: Yes, the amount that we get, and of course the food prices have gone up tremendously, so you don’t get as much for your money and then to have it stretch when you’re preparing it and eating it, it doesn’t seem to stretch as far as the kids are getting older.

Mother Smith echoed her mother’s statements about financial difficulties. To improve her nutrition, she said, “[I need] help with food costs. Money, it’s always money …” Child Smith, on the other hand, expressed a lot of frustration around his mother’s poor diet and lack of physical activity. The inflexibility in food choice purchases and lack of clear communication between family members about illness management has made him feel frustrated:

Child Smith: There’s a lotta things like she doesn’t follow the diet that diabetic’s are supposed to have, you know? Like, she drinks soda—she drinks a lotta soda and stuff … we spend like … I’m just guessing, around 40 dollars a month on soda because it supposedly like keeps her blood sugar stable.

We sensed a lot of frustration and feelings of hopelessness from the entire Smith family, with most revolving around Mother Smith’s declining health and economic barriers. Financial barriers were mentioned in almost all interviews, such as not being able to “afford better foods” (Mother Park) or try out healthy food recipes because of cost (Mother Torres).

In addition to diet, many families in the study also shared that engaging in physical activity was difficult because of issues related to resource constraints. For example, some families explained that walking in their neighbourhood and going outdoors during winter was challenging because of safety concerns and extreme weather conditions. Mother Jones, who had worked very well with Child Jones on improving dietary habits and managed to lose over 100 pounds, described that resource constraints affected their ability to engage in physical activity:

Interviewer: What sort of changes would you be interested in to make your family more active?

Mother Jones: Mm … More gas. Yeah, more gas money.

Interviewer: So what type of activities do you think your family would like to do?

Mother Jones: Go bowling. Oh, I love to go bowling, and maybe go skating … Yeah. And that’d be fun…

Interviewer: And what would get in the way to doing these?

Mother Jones: Money.

Most participants believed that one solution to improve physical activity was to go to the gym as a family, but most explained that despite really wanting to join one, they were not able to afford a gym membership.

## Discussion

To address the dearth of literature, the purpose of our study was to examine parents and their children’s perspectives on strategies used to manage their own diet and exercise and how these strategies affect other family members. Two main approaches to change emerged upon examining the family dynamics: collaborative and non-collaborative. When families used collaborative approaches, members seemed to communicate more openly to achieve their collective goal. These changes were perceived to be more successful and sustainable when family members worked together to motivate each other to eat healthier, avoid unhealthy foods, and increase physical activity. Some factors related to working more collaboratively included (a) accepting changes (especially children), (b) encouraging each other, (c) abstaining from buying and eating certain foods, (d) leading by example, and (e) mutual expectations for changes and compromise. Alternatively, when families took a non-collaborative approach to a change they described resistance and miscommunication between family members. Specific challenges that made it difficult for families to work collaboratively on changes were (a) individualistic approaches and (b) poor communication. All families struggled with lack of information and resources constraints.

The SCT framework postulates that individual characteristics (e.g., health knowledge, self-efficacy, outcome expectations) guide health behaviours (Bandura, 2004). In addition, family members interact and influence each other’s individual characteristics and environment, around diet and physical activity (i.e., family model of reciprocal determinism) (Baranowski & Hearn, 1997). The current study findings are consistent with the concept of reciprocal determinism in that family members described influencing each other’s knowledge about health, belief about their abilities to create change, and expectations of outcomes related to dietary or activity changes. Consistent with previous literature (Gadhoke et al., 2015; Laroche et al., 2008, 2009; Williams, et al., 2011), we found health approaches used by both adults and children were influenced by other family members, both in regards to diet and physical activity.

Our data suggests that when families worked more collaboratively, they felt they were more effective in meeting self-stated goals than when they worked more independently. Also, some families worked more collaboratively on physical activity but not on diet, or vice-versa. Interventions could focus on promoting collaborative approaches within the families on targeted goals and subsequently aid members to use this approach across other goals. Parents in our study initiated most of the positive health changes, which impacted their children’s behaviours. Children may be more receptive and responsive to healthy changes if they are involved in the planning process and understand the benefits of the changes. Simultaneously, as children understand the benefits of healthy eating and increased exercise, they can be supportive of positive family changes. Family-based child obesity treatment programmes that also targeted parent weight were found to be superior to only focusing on the child, suggesting that collaborative approaches to interventions might be more successful (Epstein et al., 2014). In line with the SCT model, a factor that seemed to impact positive change was that family members served as mutual supporters and were able to effectively influence one another. To this end, intervention efforts should adapt efficacious family-based approaches from child obesity treatment to address prevention efforts for both adult and child obesity.

Families working more independently may benefit from interventions that improve communication skills. Based on our findings, family members were sometimes expressing similar goals; communicating about these joint goals and finding ways to work towards them together would allow for positive changes. For instance, Children Torres expressed desire for their Mother to exercise more but Mother described that she was limited in time because she is caring for the children. Second, Family Brooks expressed that they should be consuming more vegetables and fruits but disagreed on which they liked. Third, Child Rodriguez stated that she received health information from the school but Mother Rodriguez expressed needing information on how to improve diet and physical activity. Therefore, while mothers had health-related goals, their outcome expectation (e.g., motivation phase) could be strengthened through communication with family members on family-level health goals (Baranowski & Hearn, 1997). One idea for these three cases would be to facilitate conversations between family members to either incorporate family exercise during caretaking times, make a list of vegetable and fruits that all family members enjoy, and encourage sharing health-related information with each other, respectively.

Healthcare providers and other public health practitioners have the opportunity to facilitate important conversations between family members about joint goals at an individual-level or through community-based interventions. One tool that may be used is motivational interviewing by health coaches. Motivational interviewing is a behavioural counselling approach designed to help people identify motivations for change, establish goals relevant to their values and motivations, and increase self-efficacy for achieving those goals (Miller & Rollnick, 2013). Motivational interviewing can also be helpful for families that succeed in making certain changes but not others. Specifically, motivational interviewers can facilitate important conversations about identifying strategies that are effective and highlighting the use of these specific strategies to target other behavioural changes (Miller & Rollnick, 2013).

In addition to this family approach, low-income families needed clear, culturally-relevant information and connection to appropriate resources. The families studied represent individuals at greater risk of health disparities, as studies have shown that low-income adults with diabetes are disproportionately at risk for obesity and other health problems (Conway et al., 2018). Results from this study showed that resource constraints and lack of information were major barriers for most families in this study. Families needed information, such as components of a healthy diet and resources for physical activity in the winter. In addition to basic diet information, parents needed support on techniques to introduce healthy foods to children so they do not need to rely on less successful approaches such as threats. Diet education and parenting techniques could help mothers engage in more positive conversations with their children about the benefits of a healthy diet (Davison et al., 2013), such as those seen when families used more collaborative approaches. In addition, low-income Latino adults in our study were lacking culturally-relevant information delivered in a manner appropriate to their education level and language proficiency. Latinos who are less proficient in English have more difficulty accessing health information and are less likely to trust information provided in the media (Clayman, Manganello, Viswanath, Hesse, & Arora, 2010). It is important that families are given clear information or are referred to other people and organizations capable of providing information on culturally-relevant dietary and accessible exercise resources, such as social service agencies serving monolingual Latinos.

Resource constraints also hindered families’ abilities to make changes and work together. Predictably, families in our studies expressed difficulties affording healthy foods, such as fresh vegetables and fruits. Beyond Women, Infants, and Children (WIC) vouchers and food stamps, we need to support other community efforts to bring affordable healthy food to these areas. At the family level, one idea suggested by the data is to get the family together to budget meals and shop for food together. Thus, interventions should aim at assisting family members, including older children, to plan healthy, budget-friendly meals that most people enjoy. This would be ideal to maximize resources and avoid wasting food. Collaboration is critical because child food preferences may drive purchases in low-income families who cannot afford to waste money on food that might not be eaten (Hildebrand & Shriver, 2010). More importantly, programmes should ensure that families have their basic needs (i.e., housing and food) met before a diet or physical activity intervention starts (Bruening, MacLehose, Loth, Story, & Neumark-Sztainer, 2012; Garg, Toy, Tripodis, Silverstein, & Freeman, 2015; Meyers et al., 2005; Ries et al., 2014).

### Limitations

The findings from this study fill the literature gap by being one of the few studies to examine adult-child dyads and their perspectives in implementing sustainable changes to diet and physical activity. Nevertheless, results from this study should be examined in light of some limitations. This study provided in depth detail on a small number of families. Families in this sample are Midwestern, low-income families in a small urban area and access community health centres or local food pantries. Families living in different contexts could have different experiences. Despite purposeful sampling goals, study participants included a higher number of Latino adults than African Americans and Caucasians. However, we had multiple families who were multiracial. Additionally, some families in our study had children that were too young to be interviewed, limiting our sample of families that had the added child perspective. We reached thematic saturation based on the full sample but cannot be certain in the smaller sample; however, of note, many of the themes described in this paper were echoed in the adult-only interviews (data not shown). Future studies should explore family interactions around diet and physical activity in a larger sample, including fathers, and other caretakers. Despite the limitations, our study possesses many strengths. Our community-based participatory research model allowed us to access a difficult-to-reach, low-income, population. We enriched our data by interviewing adults and children from the same family unit and considered the dynamics in our analysis. Last, by including Spanish-speaking families, we were more inclusive of an important population that is often overlooked in research and practice.

## Conclusions

Positive changes in diet and physical activity are perceived by family members to be more sustainable when they are done as a collective effort involving adults and children in the family. Therefore, interventions should focus on families as a whole, as opposed to focusing only on the adult with diabetes or obesity, take advantage of bilateral influences between parents and children, and support family members’ skills to help them work together towards a healthier lifestyle. Low-income families described strategies that they used to improve diet and engage in physical activities, despite financial constraints. Intervention efforts should reinforce these positive strategies but also provide support, financially and educationally, to promote these and other positive behaviours in other low-income families.
